# Assessment of the Immunosuppressive Potential of INF-γ Licensed Adipose Mesenchymal Stem Cells, Their Secretome and Extracellular Vesicles

**DOI:** 10.3390/cells8010022

**Published:** 2019-01-05

**Authors:** Teresa Raquel Tavares Serejo, Amandda Évelin Silva-Carvalho, Luma Dayane de Carvalho Filiú Braga, Francisco de Assis Rocha Neves, Rinaldo Wellerson Pereira, Juliana Lott de Carvalho, Felipe Saldanha-Araujo

**Affiliations:** 1 Laboratório de Farmacologia Molecular, Departamento de Ciências da Saúde, Universidade de Brasília, Brasília 70910-900, Brazil; raquelserejo@yahoo.com.br (T.R.T.S.); amanddaevelin@hotmail.com (A.É.S.-C.); luma.filiu@gmail.com (L.D.d.C.F.B.); nevesfar@gmail.com (F.d.A.R.N.); 2 Pós-graduação em Ciências Genômicas e Biotecnologia, Universidade Católica de Brasília, Brasília 70790-160, Brazil; rinaldo.pereira@catolica.edu.br (R.W.P.); julianalott@gmail.com (J.L.d.C.)

**Keywords:** mesenchymal stem cells, T-cells, conditioned medium, extracellular vesicles, TLR, INF-γ

## Abstract

There is an active search for the ideal strategy to potentialize the effects of Mesenchymal Stem-Cells (MSCs) over the immune system. Also, part of the scientific community is seeking to elucidate the therapeutic potential of MSCs secretome and its extracellular vesicles (EVs), in order to avoid the complexity of a cellular therapy. Here, we investigate the effects of human adipose MSCs (AMSCs) licensing with INF-γ and TLR3 agonist over AMSCs proliferation, migration, as well as the immunomodulatory function. Furthermore, we evaluated how the licensing of AMSCs affected the immunomodulatory function of AMSC derived-secretome, including their EVs. INF-γ licensed-AMSCs presented an elevated expression of indoleamine 2,3-dioxygenase (IDO), accompanied by increased ICAM-1, as well as a higher immunosuppressive potential, compared to unlicensed AMSCs. Interestingly, the conditioned medium obtained from INF-γ licensed-AMSCs also revealed a slightly superior immunosuppressive potential, compared to other licensing strategies. Therefore, unlicensed and INF-γ licensed-AMSCs groups were used to isolate EVs. Interestingly, EVs isolated from both groups displayed similar capacity to inhibit T-cell proliferation. EVs isolated from both groups shared similar TGF-β and Galectin-1 mRNA content but only EVs derived from INF-γ licensed-AMSCs expressed IDO mRNA. In summary, we demonstrated that INF-γ licensing of AMSCs provides an immunosuppressive advantage both from a cell-cell contact-dependent perspective, as well as in a cell-free context. Interestingly, EVs derived from unlicensed and INF-γ licensed-AMSCs have similar ability to control activated T-cell proliferation. These results contribute towards the development of new strategies to control the immune response based on AMSCs or their derived products.

## 1. Introduction

Mesenchymal Stem-Cells (MSCs) are adult multipotent cells, which present a series of important biological properties, rendering them promising tools for cell-based therapy. One of the critical properties of these cells—which has attracted the attention of the scientific community decades ago—is the ability of MSCs to control the immune response. Specifically, regarding T-cells, MSCs exert their immunomodulatory effects through a broad range of mechanisms, including cell contact, secretion of anti-inflammatory molecules and induction of regulatory T-cells [1].

Over the last few years, the field of cellular therapy using MSCs to control immune-related diseases has grown enormously [2]. The use of MSCs to treat steroid-refractory acute Graft Versus Host Disease (aGVHD) represents one of the many explored applications of MSC-based therapy. Nevertheless, despite the promising potential of MSCs, there is a consensus that the clinical response to this therapeutic modality is not homogenous [3,4,5]. Therefore, in order to enhance the immunomodulatory function of MSCs and achieve more consistent results, several strategies have been sought. Among them, the activation and licensing of MSCs [6] with inflammatory cytokines such as INF-γ [7,8] and Toll-like receptor (TLR) agonists [9,10] has been heavily investigated.

In addition to the search for MSCs licensing strategies, another strategy that has also been subject of intensive discussion is the possibility of establishing cell-free therapies, in which the effects of MSCs are guaranteed without the need for cellular infusion. Emerging data suggest that MSCs-mediated effects appear to be partly dependent on paracrine factors, such as proteins and hormones, as well as on the transference of extracellular vesicles (EVs) to target cells [11,12]. In this scenario, several researchers are investigating the effects of MSCs-derived secretome and -EVs in various contexts, in which their parental cells effectively revealed their therapeutic potential. However, information concerning the influence of MSCs licensing over the immunosuppressive potential of their secretome and derived EVs is still scarce in the literature.

With this in mind, in the present work we evaluated whether human adipose MSCs (AMSCs) licensing with INF-γ alone or in combination with Poly (I:C) (a TLR3 agonist) influenced their phenotype, proliferation, migration capacity and immunosuppressive potential. Furthermore, we collected the conditioned medium from licensed and unlicensed AMSCs and investigated their immunosuppressive capacity. Finally, we isolated, characterized and analyzed the immunomodulatory potential of licensed and unlicensed AMSCs-derived EVs.

## 2. Materials and Methods

### 2.1. AMSCs Obtention, Culture and Characterization

AMSCs (n = 3) were kindly obtained from Cellseq Solutions, as control cell batches. Each lot of these cells was obtained from a single, healthy donor after lipoaspiration procedure. The cells were cultured in Minimum Essential Medium alpha (alpha-MEM) supplemented with 15% v.v. fetal bovine serum (FBS—HyClone, Logan, UT, USA), 2 mM glutamine and 100 U/mL penicillin/streptomycin (Sigma, St. Louis, MO, USA), at 37 °C and 5% CO_2_. Medium was changed every two days and the cells were split when they reached 80–90% confluence.

AMSCs were phenotypically characterized at 3rd passage by flow cytometry (FACSVerse, BD Biosciences), using the BD Stemflow™ hMSC Analysis Kit, following manufacturer’s instructions (Pharmingen, BD Biosciences, Franklin Lakes, NJ, USA). Briefly, control and licensed AMSCs were incubated with CD105-PerCP-Cy5.5, CD73-APC, CD90-FITC, CD44-PE and with the negative cocktail markers, which included CD45/CD34/CD11b/CD19/HLA-DR antibodies, all conjugated with PE.

The cells were used between the 3rd to 6th passages for all experiments. This study was conducted with the approval of the Institutional Ethics Committee of the Faculty of Health Sciences of the University of Brasilia (35640514.5.0000.0030) and written informed consent was obtained from all participants.

### 2.2. AMSCs Licensing

For all performed experiments, we included a control group of untreated AMSCs. Licensing was performed following three different treatment strategies, which included 48h incubation of AMSCs with (i) 50 ng/mL of INF-γ; (ii) 1 µg/mL of Poly (I:C); or 50 ng/mL of INF-γ and 1 µg/mL of Poly (I:C) [13,14]. After treatment, cells were washed with PBS for three times before the beginning of the experiments.

### 2.3. AMSCs Viability and Proliferation

The effect of AMSCs licensing over cellular growth (proliferation and/or viability) was assessed by MTT [3-(4.5-dimethylthiazol-2-yl)-2,5-diphenyl tetrazolium bromide] assay, as previously described [15]. Briefly, cells were plated at 2 × 10^3^ in 96-well plates and submitted to the different licensing protocols, as described above. Then, cells were washed 3 times with PBS and received basal medium. Cell viability assay was performed at days 1, 3 and 5, counted from the end of the licensing procedure. In these time-points, 20 µL of MTT (5 mg/mL) was added in each well and the plates incubated for 3 h. After this period, MTT and medium were removed and replaced by DMSO and the plate was homogenized for 15 min. The optical density was read on a DTX 800 Series Multimode Detector (Beckman Coulter, Brea, CA, USA) at 570 nm.

### 2.4. AMSCs Migration

AMSCs migration was investigated following the licensing procedure, by wound scratch assay [16]. To this end, 2 × 10^5^ AMSCs were seeded in 6 well plates and licensed for 48 h, under the different licensing conditions. Next, AMSCs monolayers were washed with PBS and then scratched across the center of the well using a 200 µL pipette tip. AMSCs were maintained in alpha-MEM without FBS or in alpha-MEM containing 2% FBS, as a positive control. The scratch zones were photographed at 0, 12 and 24 h post-scratch using a Zeiss Primo Vert microscope equipped with a digital camera (Carl Zeiss, Heidelberg, Germany). The open area post-scratch was measured using the software ImageJ (National Institutes of Health, Bethesda, MD, USA).

### 2.5. Isolation and Activation of Peripheral Blood Mononuclear Cells (PBMCs)

Peripheral Blood Mononuclear Cells (PBMCs) were obtained from healthy volunteers by centrifugation using Ficoll-Paque PLUS (Amersham Biosciences, Uppsala, Sweden). After isolation, PBMCs were activated with 5 μg/mL of Phytohaemagglutinin (PHA, Sigma-Aldrich, St. Louis, MO, USA) and stained with 2.5 µM carboxyfluorescein succinimidyl ester (CFSE), as previously described [17,18,19]. T-cell proliferation was analyzed by Flow Cytometry (BD Biosciences, San Jose, CA, USA) after culturing PBMCs for 5 days with either AMSCs, AMSCs-conditioned medium or EVs isolated from unlicensed and licensed AMSCs, as detailed below.

### 2.6. AMSCs Co-Culture with PBMCs

The immunosuppressive effect of licensed and unlicensed AMSCs was determined by flow cytometry. Following AMSCs licensing, the medium was removed, cells were washed 3 times with PBS and immediately co-cultured with 3 × 10^5^ PHA-activated PBMCs (1:10 ratio) for 5 days [17,18]. Then, PBMCs were recovered and stained with APC-conjugated anti-CD3 antibody and assessed for T-cell proliferation.

### 2.7. PBMCs Culture with AMSCs-Derived Conditioned Medium

To analyze the effects of the medium obtained from the different strategies of AMSCs licensing over T-cell proliferation, we removed the supernatants after the AMSCs licensing protocols and added fresh RPMI medium supplemented with 10% FBS to AMSCs cultures. After 24 h, the medium was collected, centrifuged and used to culture 3 × 10^5^ PBMCs activated with 5 μg/mL PHA [19]. In the 5th day of culture, PBMCs were collected, stained with anti-CD3 and T-cell proliferation determined by Flow Cytometry.

### 2.8. Vascular Cell Adhesion Protein 1 (VCAM-1) and Intercellular Adhesion Molecule 1 (ICAM-1) Expression on AMSCs

Considering the importance of the adhesion molecules in MSCs-mediated immunosuppression, we investigated the expression of ICAM-1 (CD54) and VCAM-1 (CD106) in licensed and unlicensed AMSCs, using monoclonal antibodies. Briefly, after licensing, cells were washed with PBS, harvested and stained with anti-CD54 (conjugated with allophycocyanin—APC), anti-CD106 (conjugated with fluorescein isothiocyanate—FITC) or isotype controls (eBioscience, San Diego, CA, USA). After incubation with the antibodies, the cells were analyzed by Flow Cytometry.

### 2.9. EVs Isolation and Characterization

After observing that the most suppressive conditioned medium was obtained from INF-γ licensed AMSCs, we isolated EVs from this group, as well as from unlicensed AMSCs, in order to assess their capacity of controlling activated T-cell proliferation. Briefly, AMSCs were cultured until confluence in 75 cm^5^ flasks containing 10 mL basal medium supplemented with 10% v.v. of microvesicles-free FBS. When AMSCs reached confluence they were licensed for 24 h, the supernatant was collected and, EV isolation was immediately performed using total exosome isolation reagent (Invitrogen, Life Technologies, Carlsbad, CA, USA), as described by the manufacturer. Cell culture medium was centrifuged at 2000 *g* for 30 min to remove cellular debris, mixed with 5 mL of total exosome isolation reagent and incubated at 4 °C overnight. After incubation, samples were centrifuged at 10,000× *g* for 1 h and the pellets containing EVs were resuspended in PBS. Protein concentration was determined by Bradford method [20].

EVs were initially characterized according to average diameter using Zetasizer Nano ZS (Malvern Instruments, Malvern, UK), following to manufacturer’s instructions. EVs diameter was also determined by transmission electron microscopy (TEM). For this, 5 µL of EVs samples were mounted on formvar copper grids and fixed in Karnovsky EM fixative solution (2% formaldehyde and 2.5% glutaraldehyde in 0.1 mol/L sodium cacodylate buffer, pH 7.4). Samples were then negatively stained using 2% aqueous phosphotungstic acid (PTA), examined and photographed with a JEOL JEM1011 transmission electron microscope operating at 80 kV.

EVs were also phenotypically characterized by flow cytometry using CD105-PerCP-Cy5.5 and CD90- FITC antibodies. For this, EVs were coupled with 4-µm-diameter aldehyde/sulfate latex beads and then blocked by incubation with FBS. EVs-coated beads were washed three times in PBS and resuspended in 50 μL of PBS. Next, beads were incubated with the aforementioned antibodies and analyzed by Flow Cytometry.

### 2.10. Immunosuppressive Effects of AMSCs-Derived EVs

To access the immunosuppressive potential of AMSCs-derived EVs, 3 × 10^5^ PBMCs were activated with 5 μg/mL of PHA and cultured for 5 days with 0.25, 0.75 or 3.0 μg of EVs isolated from both unlicensed and INF-γ licensed AMSCs [21]. After this period, PBMCs were collected, stained with anti-CD3 and T-cell proliferation was determined by Flow Cytometry.

### 2.11. RNA Isolation and Real-Time PCR

Gene expression analysis was performed in unlicensed and licensed AMSCs, as well as their EVs. RNA samples were obtained using Trizol reagent. RNA amount and quality were determined by NanoDrop 1000 spectrophotometer (Wilmington, DE, USA). One microgram of RNA was converted to single-stranded cDNA, using the High Capacity Kit (Applied BioSystems, Foster City, CA, USA) according to manufacturer’s recommendations. Real-time PCR was performed using TaqMan probes and MasterMix (Applied BioSystems, Foster City, CA, USA), following manufacturer’s instructions.

Real time PCR for TNF (Hs01113624), TGF-β (Hs00998133), IDO (Hs00984148), Galectin-1 (Hs00355202), IL-1β (Hs00174097) and IL-10 (Hs00961622) was run in duplicates and the relative fold change obtained by the 2^−ΔΔCt^ method [22]. GAPDH was used as internal reference. The median Ct values of unlicensed AMSCs and their EVs were used as reference. Cycling parameters were 95 °C for 10 min followed by 40 cycles of 95 °C for 15 s and 60 °C for 1 min.

### 2.12. Statistical Analysis

The results are presented as mean ± SEM of three independent experiments. Statistical analyses were performed using Prism 7 software (GraphPad Software Inc., San Diego, CA, USA). Statistical significance was calculated using *t*-test analyses, considering *p* < 0.05.

## 3. Results

### 3.1. INF-γ and/or Poly (I:C) Licensing Maintain AMSCs Phenotype

AMSCs had a typical MSCs immunophenotype, with positive expression of CD44, CD73, CD90 and CD105 markers and negative expression of CD34, CD45, CD11b, CD19 and HLA-DR. We also investigated if the licensing treatments with INF-γ and/or Poly (I:C) would alter AMCSs immunophenotype, however, the phenotypic pattern was maintained in all samples, regardless of the licensing strategy adopted (Supplementary Figure S1)

### 3.2. INF-γ and/or Poly (I:C) Licensing did not Influence AMSCs Proliferation

**Figure 1:**
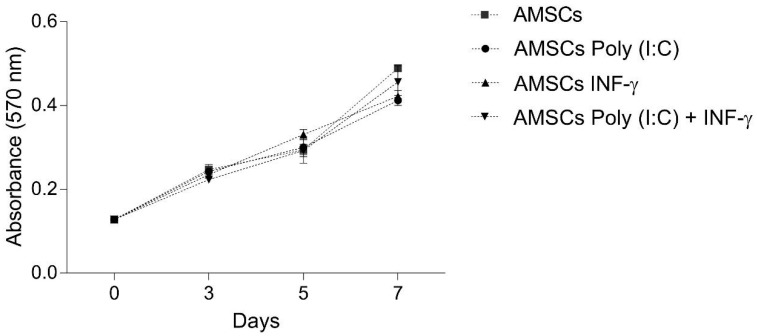
Proliferative capacity of licensed and unlicensed AMSCs. Control AMSCs, AMSCs licensed with 1 µg/mL of Poly (I:C), AMSCs licensed with 50 ng/mL of INF-γ; and AMSCs licensed with 50 ng/mL of INF-γ and 1 µg/mL of Poly (I:C) were cultured and cell proliferation was assessed by MTT in the days 3, 5 and 7 of the culture. No difference of proliferation/viability was observed among the groups. Values represent the means ± SEM. Three independent experiments were performed.

Considering that MSCs immunosuppressive effects are dose-dependent, we evaluated if INF-γ and/or Poly (I:C) licensing could modulate AMSCs proliferation. Obtained results revealed that none of the licensing strategies tested modified AMSCs proliferation (Figure 1).

### 3.3. INF-γ and/or Poly (I:C) Licensing Did not Alter AMSCs Migration

**Figure 2:**
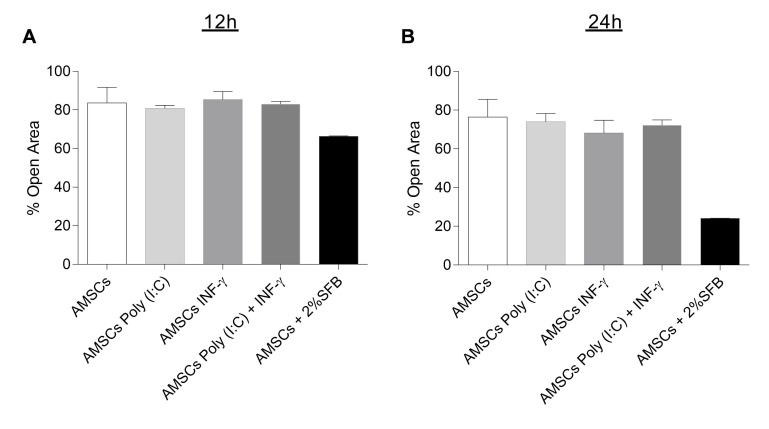
Migratory potential of licensed and unlicensed AMSCs. (**A**) Wound scratch assays for unlicensed AMSCs, as well as licensed AMSCs treated with either 1 µg/mL Poly (I:C), 50 ng/mL INF-γ or with both 50 ng/mL of INF-γ and 1 µg/mL of Poly (I:C). Confluent cells were wounded by a scratch with a pipette tip and cell migration was assessed under the microscope at 12 h and 24 h (**B**). Results are presented as mean ± SEM of three independent experiments.

Control and licensed AMSCs were investigated regarding their migration potential by wound scratch assay, but, once again, we observed that the licensing regimes did not affect the migratory behavior of AMSCs after 12 h and 24 h (Figure 2).

### 3.4. INF-γ Enhances AMSCs-Mediated Immunomodulation

**Figure 3:**
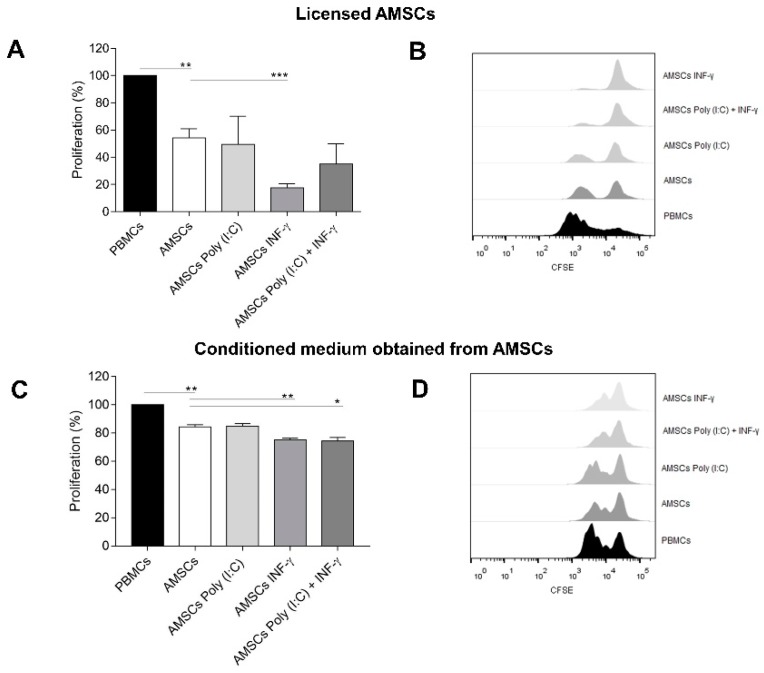
Immunosuppressive capacity of licensed AMSCs and their conditioned medium. (**A**) unlicensed AMSCs, as well as AMSCs licensed with either 1 µg/mL of Poly (I:C), 50 ng/mL of INF-γ or with both 50 ng/mL of INF-γ and 1 µg/mL of Poly (I:C) were cocultured with PHA-activated PBMCs (1:10 ratio) and T-cell proliferation was determined by Flow Cytometry after 5 days. Results are presented as mean ± SEM of three independent experiments; (**B**) Representative CFSE histograms of one AMSCs sample investigated; (**C**) After AMSCs licensing, medium were discarded, cells were washed 3 times with PBS and fresh medium was added to each condition. After 24 h, the AMSCs conditioned medium (from licensed and unlicensed samples) were harvested and used to culture PHA-activated PBMCs, so that T-cell proliferation could be analyzed after 5 days of treatment. Results are presented as mean ± SEM of three independent experiments; (**D**) Representative CFSE histograms from conditioned medium from one AMSCs sample investigated. * *p* < 0.05. ** *p* < 0.01. *** *p* < 0.001.

Consistent with published literature [23], our data revealed that AMSCs co-culture markedly decreased activated T-cell proliferation (*p* = 0.0003). Wondering whether licensed AMSCs presented different immunosuppression effect compared to unlicensed cells, we performed the same co-culture assay with licensed AMSCs. Interestingly, INF-γ licensed AMSCs presented significantly higher capacity to inhibit activated T-cell proliferation (*p* = 0.003). On the other hand, licensing with Poly (I:C) alone did not influence AMSCs-mediated immunosuppression capacity. Even though we did not detect a statistically significant difference, the licensing of AMSCs with both Poly (I:C) and INF-γ increased their suppressive potential in 35% (mean), compared to unlicensed AMSCs (Figure 3A,B).

### 3.5. Conditioned Medium from INF-γ Licensed AMSCs Has Increased Capacity to Control the T-Cell Response

Aiming to further investigate the possible use of the AMSCs secretome in a cell free perspective, we isolated the conditioned medium of licensed and unlicensed AMSCs and investigated their immunosuppressive potential. We observed that the conditioned medium obtained from unlicensed AMSCs suppress T-cell proliferation (*p* = 0.004). The conditioned medium derived from AMSCs licensed with Poly (I:C) and INF-γ presented a slightly increased capacity to suppress T-cell proliferation (*p* = 0.01) compared to conditioned medium obtained from unlicensed AMSCs (*p* = 0.01). Importantly, the conditioned medium isolated from INF-γ licensed AMSCs showed the highest capacity to inhibit activated T-cell proliferation (*p* = 0.005) among tested groups (Figure 3C,D).

### 3.6. INF-γ Enhances ICAM-1 Expression on AMSCs

**Figure 4:**
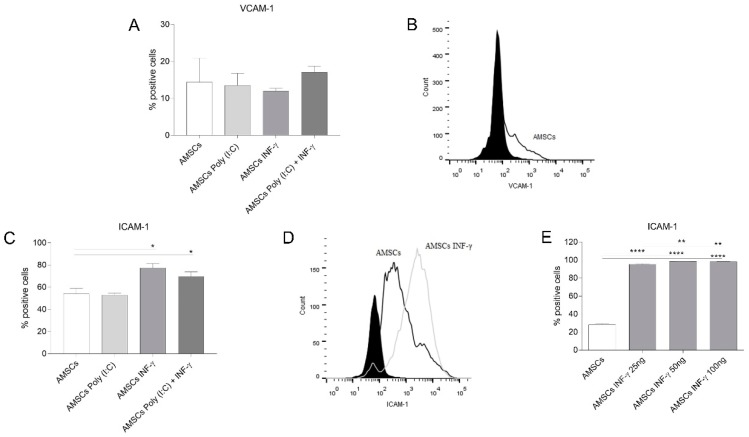
VCAM-1 and ICAM-1 expression of licensed and unlicensed AMSCs. (**A**) unlicensed AMSCs, as well as AMSCs licensed with either 1 µg/mL of Poly (I:C), 50 ng/mL of INF-γ or with both 50 ng/mL of INF-γ and 1 µg/mL of Poly (I:C), were used to assess VCAM-1 expression by Flow Cytometry. Results are presented as mean ± SEM of three independent experiments; (**B**) Representative histogram showing VCAM-1 expression of one sample of unlicensed AMSCs;. (**C**) unlicensed AMSCs, as well as AMSCs licensed with either 1 µg/mL of Poly (I:C), 50 ng/mL of INF-γ or with both 50 ng/mL of INF-γ and 1 µg/mL of Poly (I:C) were also used to investigate ICAM-1 expression by Flow Cytometry. Results are presented as mean ± SEM of three independent experiments; (**D**) The influence of INF-γ over ICAM-1 expression on AMSCs was confirmed using three different concentrations of this inflammatory factor. (**E**) ICAM-1 expression on unlicensed AMSCs and AMSCs licensed with 25, 50 and 100 ng/mL of INF-γ. * *p* < 0.05, ** *p* < 0.01, **** *p* < 0.0001.

Considering the importance of adhesion molecules in the context of contact-dependent MSCs-immunosuppression, we investigated the expression of VCAM-1 and ICAM-1 on licensed and unlicensed AMSCs. Flow cytometry data of unlicensed AMSCs presented a mean expression of VCAM-1 and ICAM-1 of 14.38 and 54%, respectively (Figure 4A,C). While none of the licensing strategies altered the expression of VCAM-1 on AMSCs (Figure 4A,B), ICAM-1 expression was increased in INF-γ licensed AMSCs (*p* = 0.01), as well as in INF-γ and Poly (I:C) licensed cells (*p* = 0.03) (Figure 4C,D). Interestingly, when we licensed AMSCs using 25, 50 and 100 ng/mL of INF-γ, we noticed that the effect of this inflammatory factor over the AMSCs expression of ICAM-1 is increased (*p* < 0.01) between lower (25 ng/mL; mean value 95.4%) and higher concentrations (50/100 ng/mL; mean values 98.3/98.2%) (Figure 4E).

### 3.7. EVs Characterization

**Figure 5:**
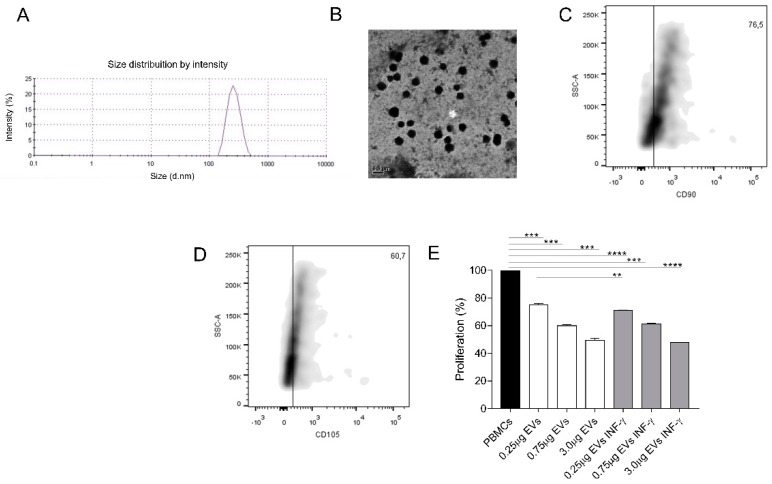
Characterization of EVs isolated from unlicensed and INF-γ licensed AMSCs and their capacity to control T-cell proliferation. (**A**) EVs average size estimated using Zetasizer Nano ZS; (**B**) Transmission electron microscopy of EVs (representative image of one unlicensed sample); CD90 (**C**) and CD105 (**D**) expression of EVs isolated from unlicensed AMSCs were determined by Flow Cytometry; (**E**) EVs isolated from unlicensed and INF-γ licensed AMSCs were quantified according to their protein concentration by Bradford assay and used in different concentrations (0.25, 0.75 and 3.0 µg) to treat PHA-activated PBMCs, in order to access their immunosuppressive capacity. Results are presented as mean ± SEM of three independent experiments. ** *p* < 0.01, *** *p* < 0.001 **** *p* < 0.0001.

EVs isolated from unlicensed AMSCs showed the mean size of 262.4 nm, as determined by Zetasizer Nano ZS measurement (Figure 5A) and TEM (Figure 5B). INF-γ licensed AMSCs did not present significant differences regarding average size, which was 264.2 nm (data not shown). EVs characterization was also performed by Flow Cytometry, being that the isolated EVs were immunophenotypically characterized and showed positive expression of MSCs markers CD90 (76.5%) and CD105 (60.7%) (Figure 5C,D).

### 3.8. AMSCs-Derived EVs Present Immunosuppressive Potential

After characterization, we evaluated if EVs derived from unlicensed and INF- γ licensed AMSCs presented immunoregulatory potential of inhibiting activated PBMCs proliferation. Notably, PBMCs incubation with 0.25, 0.75 and 3.0 μg of unlicensed AMSCs-derived EVs successfully suppressed activated T-cell proliferation (*p* = 0.0005; *p* = 0.0002 and *p* < 0.0003, respectively). Likewise, PBMCs incubation with 0.25, 0.75 and 3.0 μg of INF-γ licensed AMSCs also suppressed T-cell proliferation (*p* < 0.0001; *p* = 0.0002 and *p* < 0.0001, respectively). Importantly, even though both groups effectively promoted immunosuppression at all concentrations tested, when used at 0.25 μg, we detected a slight increase in the suppressive potential of EVs isolated from INF-γ licensed AMSCs compared to EVs from unlicensed AMSCs (*p* = 0.004) (Figure 5E).

### 3.9. Expression of Inflammatory Transcripts in Licensed and Unlicensed AMSCs and in Their EVs

**Figure 6:**
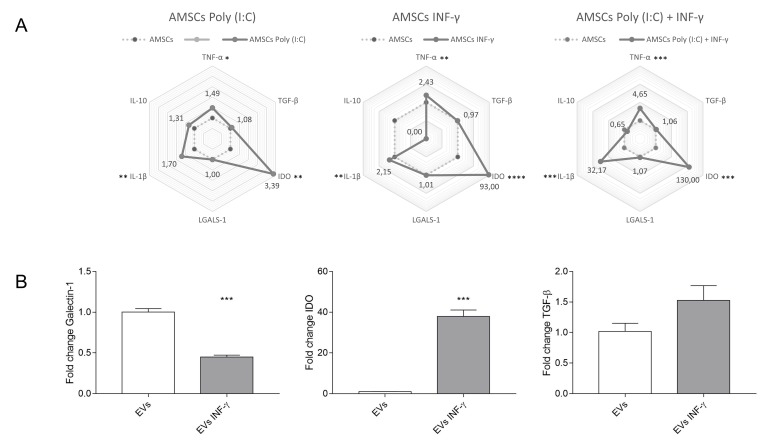
Gene expression analysis of selected transcripts in unlicensed and licensed AMSCs. (**A**) Radial plot demonstrating the differences in overall transcripts between unlicensed AMSCs, as well as AMSCs licensed with 1 µg/mL of Poly (I:C), AMSCs licensed with 50 ng/mL INF-γ and AMSCs licensed with both 50 ng/mL of INF-γ and 1 µg/mL of Poly (I:C). Solid vertices represent the mean fold change of individual transcripts. Median Ct value of unlicensed AMSCs was used as a reference. Results are presented as the mean of three independent experiments; (**B**) Expression of Galectin-1, IDO, IL-10 and TGF-β transcripts in EVs isolated from unlicensed and INF-γ licensed AMSCs. To analyze IDO expression in EVs, the CT value of EVs obtained from unlicensed AMSCs was arbitrarily defined as 40. *** *p* < 0.001.

Gene expression of TNF, TGF-β, IDO, Galectin-1, IL-1β and IL-10 was assessed in licensed and unlicensed AMSCs by Real Time PCR. Compared to unlicensed AMSCs, INF-γ treatment increased the expression of TNF (*p* = 0.002), IL-1β (*p* = 0.001) and IDO (*p* < 0.0001), the latter with more intensity. Interestingly, this licensing protocol abrogated IL-10 transcription. AMSCs licensing with Poly (I:C) induced a higher expression of TNF (*p* = 0.04), IL-1β (*p* = 0.008) and IDO (*p* = 0.001), as well. Interestingly, AMSCs licensing with both Poly (I:C) and INF-γ induced the most intense transcriptional differences compared to unlicensed AMSCs, leading to the highest expression of TNF (*p* = 0.0003), IL-1β (*p* = 0.0003) and IDO (*p* = 0.001). Of note, none of the tested strategies of AMSCs licensing influenced Galectin-1 and TGF-β expression (Figure 6A). We also assessed the transcriptional levels of TGF-β, IDO, Galectin-1 and IL-10 in EVs isolated from unlicensed and INF-γ licensed AMSCs. Interestingly, EVs from INF-γ licensed AMSCs showed decreased expression of Galectin-1 transcript (*p* = 0.0002). However, IDO was detected only in EVs isolated from INF-γ licensed AMSCs (*p* = 0.0001). No statistically significant difference was detected regarding TGF-β expression between groups (Figure 6B). IL-10 expression was not detected in the analyzed EV transcripts.

## 4. Discussion

In the present study, we have demonstrated that the licensing of AMSCs with INF-γ and/or Poly (I:C) maintain the classic AMSCs phenotypic pattern and does not significantly alter AMSCs proliferative capacity and migratory behavior. Importantly, though, our data reveal that INF-γ licensing markedly induces AMSCs to produce higher levels of IDO, increased the expression of ICAM-1 adhesion molecule and potentializes the capacity of licensed cells to suppress activated T-cell proliferation, compared to unlicensed counterparts. On the other hand, we have also clearly demonstrated that under a perspective of a cell free therapy, the strategy of licensing of AMSCs with INF-γ was effective in promoting immunoregulatory advantages when compared to unlicensed cell samples. Finally, our data reveal that AMSCs present a constitutive potential to inhibit activated T-cell proliferation and to secrete biologically active EVs, which harbor the capacity of effectively controlling T-cell response.

Several processes contribute for MSCs immunoregulatory potential. For instance, to exert their immunoregulatory effects with the greatest potential, MSCs must survive and reach sites of injury. However, it is currently established that only a small number of infused cells can achieve this goal, following stem cell therapy. Therefore, the search for strategies capable of enhancing the suppressive capacity of MSCs is paramount to guarantee the efficacy and commercial viability of such therapy [24,25]. In this sense, among the several strategies under investigation to boost MSC therapy efficacy, lie the licensing protocols. According to this rationale, it may be possible to stimulate MSCs to boost their pro-survival and immunomodulation properties, by treating them with specific molecules prior to treatment. Several licensing strategies have been tested so far, such as the treatment of MSCs with INF-γ and Poly (I:C). According to previous reports, INF-γ signaling did not improve the migratory capacity of bone marrow and cord blood-derived MSCs [26]. Accordingly, we have not found any effect of INF-γ regarding the migratory capacity of AMSCs. Considering the role of TLR signaling in MSCs migration, it has been showed that Poly (I:C) stimulation enhanced the migratory capacity of MSCs derived from bone marrow [27]. In contrast to previous reports, we have not noticed any impact of TLR3 signaling over AMSCs migratory behavior. In part, these discrepant results can be explained by the fact that MSCs present different migratory capacity depending on their source of obtention, as described recently [28] and probably also differ in the response to variable stimuli. This hypothesis may be subsidized by the observation that circulating MSCs are derived from bone marrow [29,30], suggesting that MSCs from other tissues may not respond to the same migration stimuli similarly.

The development of strategies to enhance MSCs proliferation has particular relevance considering that their immunomodulatory effects are dose-dependent [31]. In this context, data concerning the influence of TLR3 and INF-γ signaling on human AMSCs proliferation are markedly scarce in the literature. Our results showed that the licensing of these cells with TLR3 and/or INF-γ did not change AMSCs proliferation. In agreement with our observation, others also failed to detect any influence of TLR3 signaling in AMSCs proliferative capacity [32,33]. Long term stimulation with INF-γ, on the other hand, has been documented to reduce the proliferation of bone marrow derived MSCs [34]. More recently, it has been demonstrated that 5 days stimulation of bone marrow-derived MSCs with low a concentration of INF-γ (i.e., 0.1 ng/mL), actually increased cell proliferation but also that, when stimulated with higher levels of INF-γ (i.e., 10 ng/mL), cell proliferation was markedly compromised. In this conflicting scenario, it is important to note that, in contrast to our experimental design, MSCs were continuously maintained in the presence of INF-γ in the studies mentioned above [35].

Since the demonstration that murine MSCs licensing with INF-γ could completely prevent GVHD mortality [36], several efforts have been made to better understand the effects of this inflammatory factor over MSCs-mediated immunomodulation. Here, we have shown that AMSCs licensing with INF-γ was indeed an effective strategy to enhance AMSCs’ capacity to control activated T-cell proliferation. Interestingly, this licensing protocol increased the expression of ICAM-1 protein expression and of IDO transcript levels in AMSCs, both of which have important roles in the immunomodulation exerted by these cells [37,38].

Another important strategy that has been explored to enhance MSCs-mediated immunomodulation is the stimulation of such cells with TLR3 agonists. In this sense, the results presented in the literature indicate that the positive effects of this strategy seem to be inconsistent and dependent on the MSCs source under investigation. While TLR3 signaling improves the immunosuppressive effects of MSCs isolated from the umbilical cord [39] and bone marrow [40], we have not noticed any influence on AMSCs. Accordingly, Lombardo and colleagues reported that TLR3 signaling in AMSCs did not influence their immunoregulatory phenotype [33]. In our hands, we have also explored the effects of AMSCs licensing with a combination of INF-γ and Poly (I:C). Surprisingly, though, we have observed only a slight reduction in lymphocyte PHA-induced proliferation. Besides, even though the combined INF-γ and Poly (I:C) licensing strategy significantly enhanced ICAM-1 and IDO expression, it also promoted a substantial increase in TNF and IL-1b transcript levels, two critical proinflammatory factors [41,42]. We noticed that AMSCs licensing with INF-γ abrogated IL-10 transcription, in contrast to the licensing with Poly (I:C), where IL-10 transcription was stimulated. Importantly, IL-10, as well as PGE2, TGF-β, IGF and HLA-G5, play an important role to generate Tregs, which enhance MSCs-mediated immunosuppression [43,44,45,46].

Importantly, the conditioned medium from unlicensed AMSCs showed capacity to control T-cell proliferation. In accordance with this data, Matula and colleagues also showed that conditioned medium from unlicensed AMSCs has immunosuppressive potential [47]. More importantly, we demonstrated that this immunomodulatory potential can be enhanced by INF-γ licensing. Given that AMSCs licensing with INF-γ was able to potentialize the intrinsic capacity of AMSCs conditioned medium to control activated T-cell proliferation, we continued our investigation with the isolation of EVs from this group and from unlicensed AMSCs, considering that the analysis of such EVs could provide new insights to the field of cell free technologies. In fact, the conditioned medium and the EVs isolated from MSCs are currently being explored for the most varied applications and already showed promising effects in animal models of acute myocardial infarct, as well as lung, kidney and brain injuries [48]. Interestingly, isolated EVs showed positive expression of MSCs markers and an average size compatible with previous results reported by Blazquez et al. [21]. These authors also demonstrated that EVs derived from AMSCs are able to control T-cell proliferation. In addition, we showed that both EVs isolated from unlicensed and INF-γ licensed AMSCs showed potential to significantly suppress activated T-cell proliferation and that in lower EVs concentrations, INF-γ licensed group presented a greater immunoregulatory effect. A previous work failed to detect any differences regarding the immunosuppressive potential of EVs derived from unlicensed and INF-γ licensed MSCs [49]. However, it is important to point that, despite being performed with murine bone marrow MSCs, the licensing strategy used in this study was performed with lower concentrations of INF-γ. In order to investigate molecular mechanisms that could be involved in the immunosuppressive effects observed, we investigated the presence of anti-inflammatory factors in EVs from unlicensed and INF-γ licensed AMSCs. Importantly, we demonstrated that these EVs carry transcripts of anti-inflammatory genes, involved in MSCs mediated immunoregulation, such as galectin-1 [50] and TGF-B [44]. IDO transcripts were detected only in EVs derived from INF-γ licensed AMSCs, however, we did not observe any striking immunosuppressive advantage in this group, suggesting that other anti-inflammatory players may have more significant roles in the immunosuppressive potential of AMSCs-derived EVs. The demonstration that both AMSCs conditioned medium and derived EVs are immunologically active has particular relevance, especially when taking into consideration the significant advantages of cell-free components compared to their cellular counterparts. For instance, cell-free material obtention, handling and production yields are more attractive compared to cell products, as well as the elimination of the risks associated to cellular infusion.

## 5. Conclusions

In summary, in the present work, we sought to comprehensively investigate the immunosuppressive potential of AMSCs under different licensing strategies, considering their direct use, as well as their conditioned medium and derived EVs. Our results clearly show that the licensing of AMSCs with INF-γ increases their immunoregulatory potential, which is accompanied by an increase in the expression of IDO and ICAM. Additionally, we have shown that conditioned medium obtained from INF-γ licensed AMSCs display a higher capacity to control T-cell proliferation compared to conditioned medium from unlicensed counterparts. Finally, our data clearly demonstrated that both EVs isolated from unlicensed and INF-γ licensed AMSCs are also capable to control the T-cell proliferation. These results contribute to the better elucidation of the suppressive potential of AMSCs and their products, serving as the basis for the development of new therapeutic approaches to control the immune response.

## Supplementary Materials

The following is available online at http://www.mdpi.com/2073-4409/8/1/22/s1. Figure S1: Immunophenotypic characterization of AMSCs.
