# Supplementary material for: Assessment of the Immunosuppressive Potential of INF-γ Licensed Adipose Mesenchymal Stem Cells, Their Secretome and Extracellular Vesicles
Source: Cells. 2019 Jan 5;8(1):22. doi: 10.3390/cells8010022 (PMC6356584; doi:10.3390/cells8010022)
Supplement: Supplementary file 1 [file cells-08-00022-s001.pdf]

cells MDPI

# Assessment of the Immunosuppressive Potential of INF-γ Licensed Adipose Mesenchymal Stem Cells, Their Secretome and Extracellular Vesicles

**Teresa Raquel Tavares Serejo [1], Amandda Évelin Silva-Carvalho [1], Luma Dayane de Carvalho Filiú Braga [1], Francisco de Assis Rocha Neves [1], Rinaldo Wellerson Pereira [2], Juliana Lott de Carvalho [2] and Felipe Saldanha-Araujo [1,*]**

[1] Laboratório de Farmacologia Molecular, Departamento de Ciências da Saúde, Universidade de Brasília, Brasília 70910-900, Brazil; raquelserejo@yahoo.com.br (T.R.T.S.); amanddaevelin@hotmail.com (A. É.S.-C.); luma.filiu@gmail.com (L.D.d.C.F.B.); nevesfar@gmail.com (F.d.A.R.N.)

[2] Pós-graduação em Ciências Genômicas e Biotecnologia, Universidade Católica de Brasília, Brasília 70790-160, Brazil; rinaldo.pereira@catolica.edu.br (R.W.P.); julianalott@gmail.com (J.L.d.C.)

* Correspondence: felipearaujo@unb.br; Tel./Fax: +55-61-3107-2008

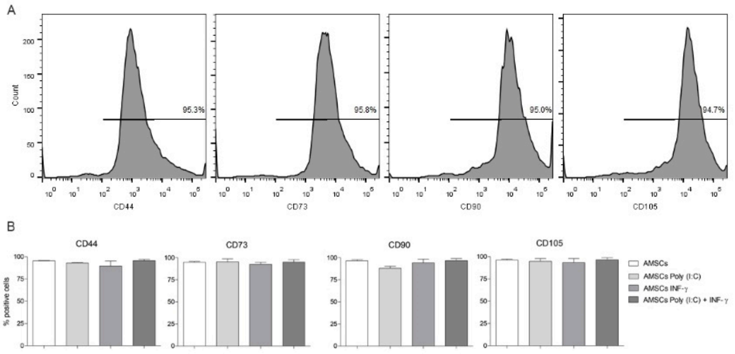


**Figure S1.** Immunophenotypic characterization of AMSCs.
